# Supplementary material for: Automated 3D Axonal Morphometry of White Matter
Source: Sci Rep. 2019 Apr 15;9:6084. doi: 10.1038/s41598-019-42648-2 (PMC6465365; doi:10.1038/s41598-019-42648-2)
Supplement: Supplementary file 1 — Supplementary Information [file 41598_2019_42648_MOESM1_ESM.pdf]

Automated 3D Axonal Morphometry of White Matter

Ali Abdollahzadeh[1], Ilya Belevich[2], Eija Jokitalo[2], Jussi Tohka[1+], and Alejandra Sierra[1*+]

[1]Biomedical Imaging Unit, A.I.Virtanen Institute for Molecular Sciences, University of Eastern Finland, Kuopio, Finland

[2]Electron Microscopy Unit, Institute of Biotechnology, University of Helsinki, Helsinki, Finland

*alejandra.sierralopez@uef.fi

+These authors contributed equally to this work

Supplementary Information

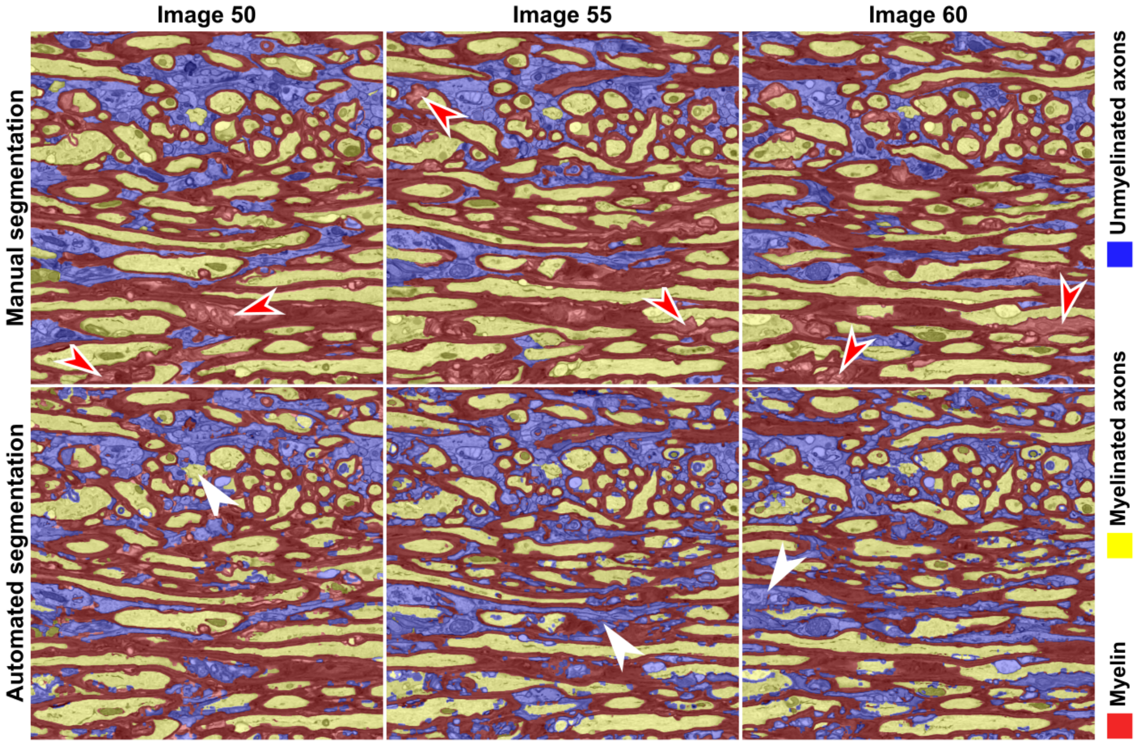

Figure S1. Manual and automated segmentations of three images from the contralateral corpus callosum of Sham-1 dataset, represented with a 3 labels map of myelin and intra-axonal space of myelinated and unmyelinated axons. As in Fig. 2, the red arrowheads indicate delamination in the myelin sheaths. These substructures were annotated as myelin in the manual annotation, while the automated segmentation excluded the myelin delamination from the myelin-labeled structures. White arrowheads point at segmentation errors where the membrane of unmyelinated axons was poorly resolved.

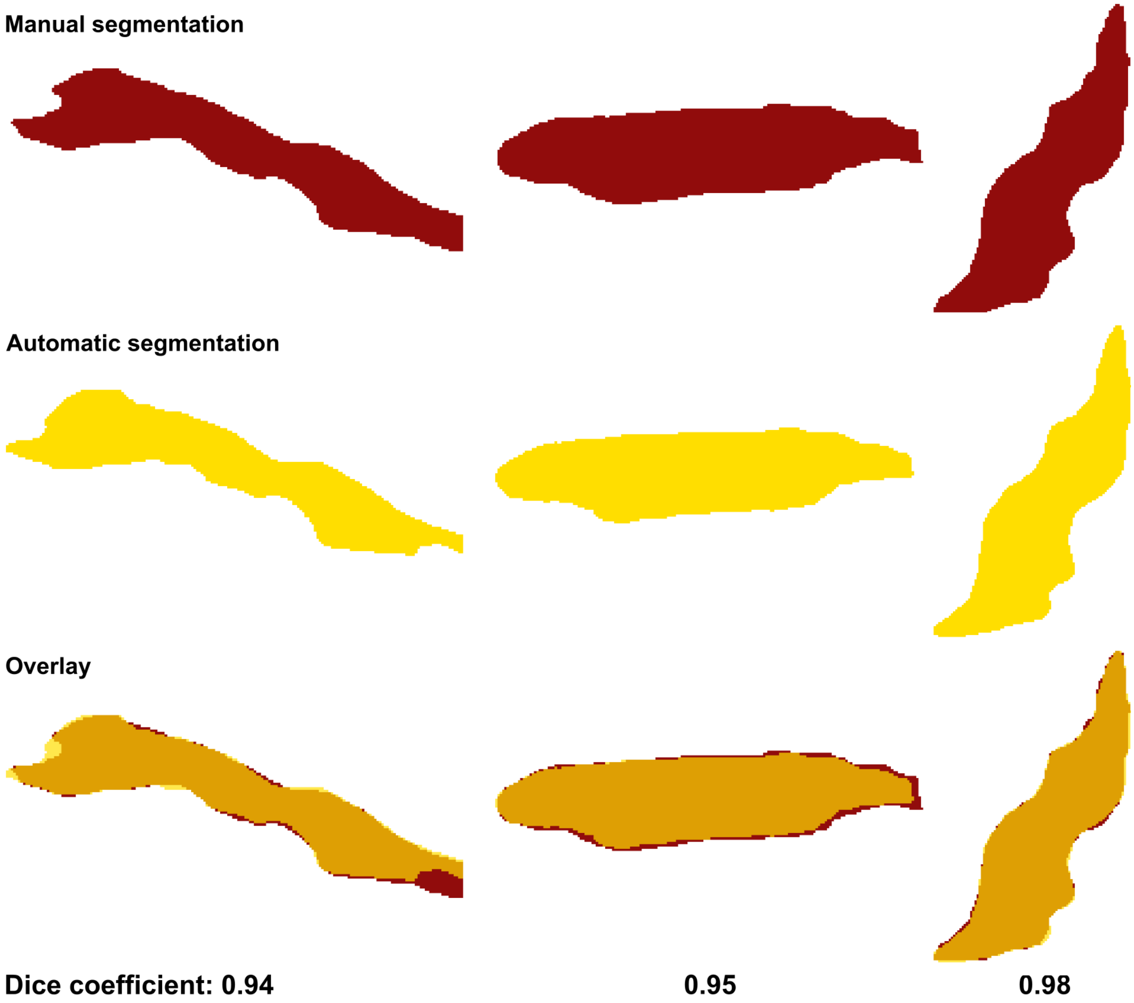

Figure S2. Comparison of manual and automated segmentation. Three representative segments of myelinated axons selected from contralateral corpus callosum of Sham-1 dataset. The Dice coefficients are sensitive to minor displacements in the location of boundaries.

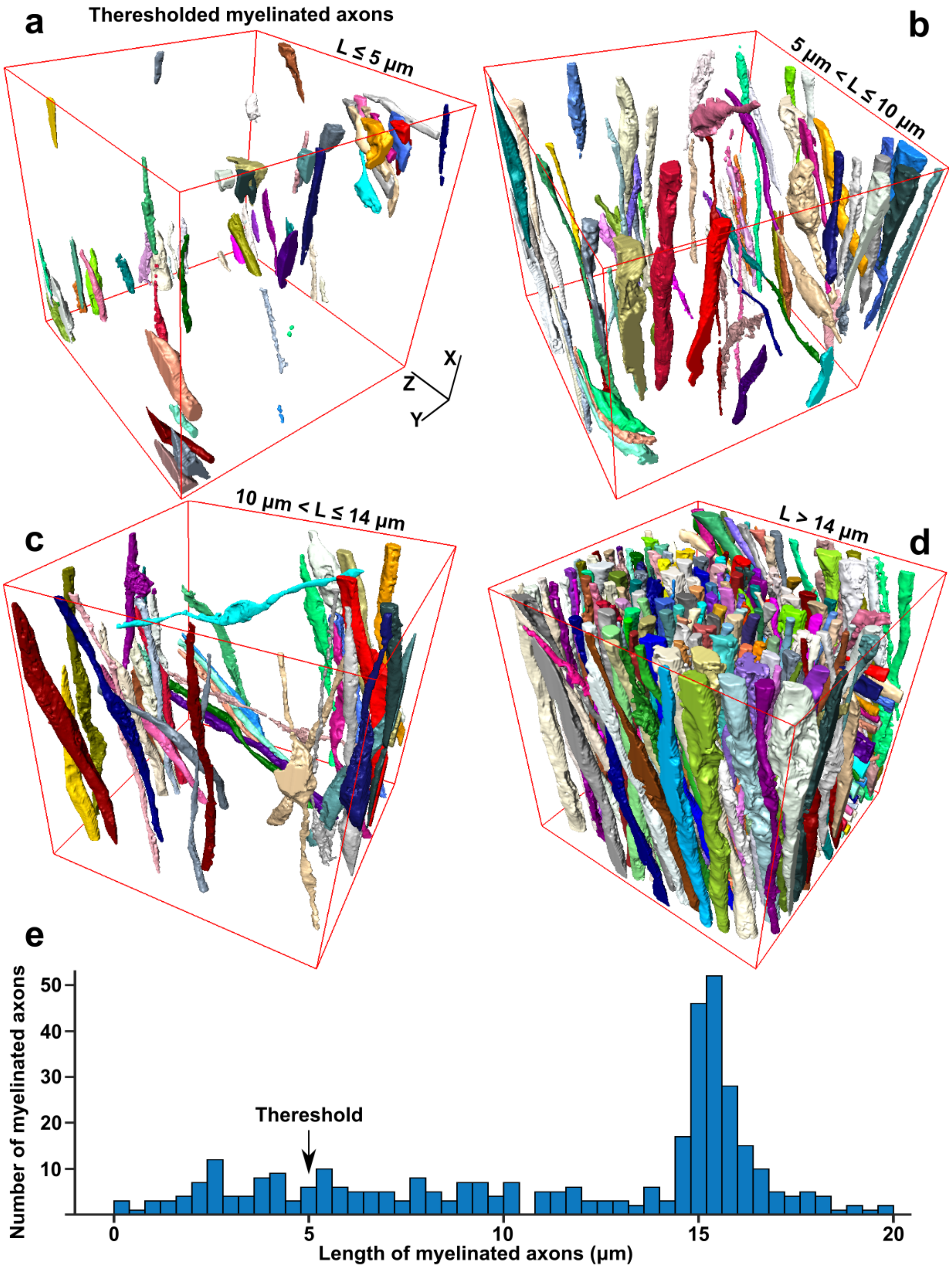

Figure S3. Evaluation of split and merge errors in segmentation of myelinated axons in contralateral corpus callosum of Sham-1 dataset. (a-d) 3D rendering of myelinated axons represented based on their length (L). Short axons ($L \leq 5\,\mu m$) traversed the corners of the SBEM volume, and they were not the result of split error. (**e**) Distribution of the length of myelinated axons. We thresholded myelinated axons at 5 µm to exclude myelinated axons traversing partially the SBEM volumes from later quantification. About 17% of myelinated were shorter than the threshold.

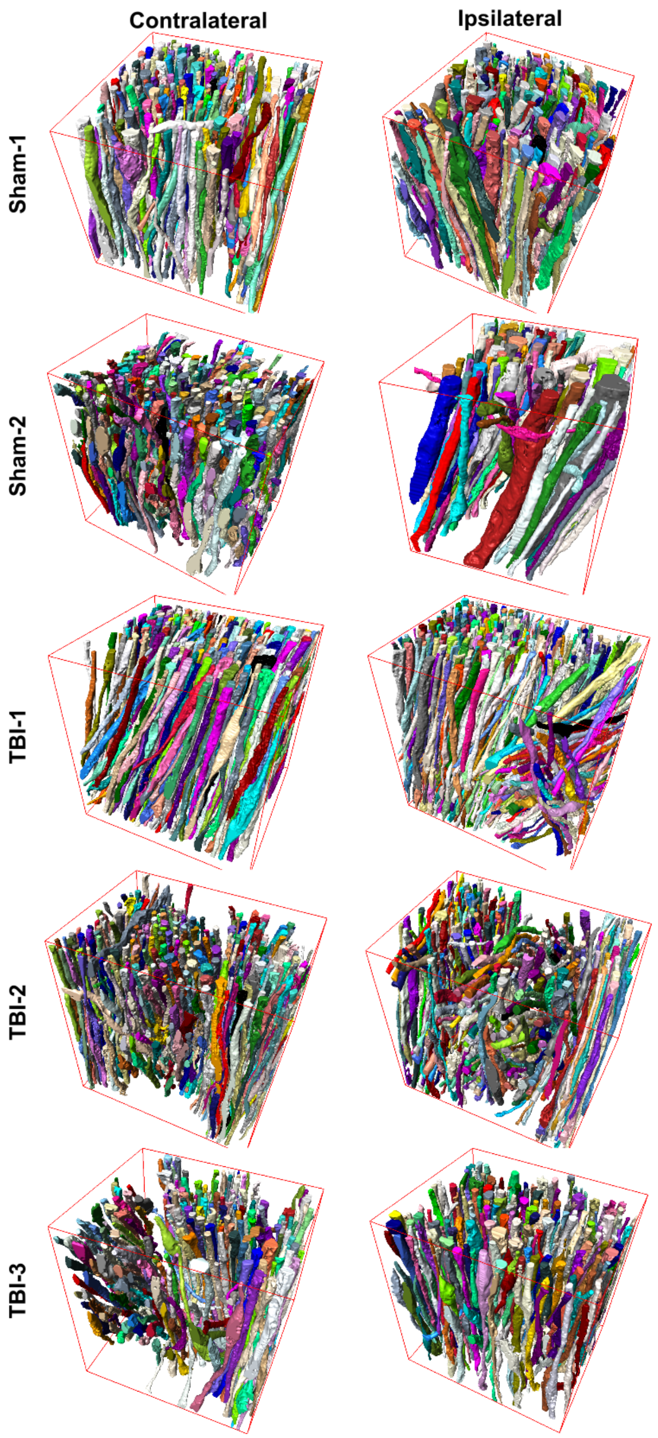

Figure S4. 3D rendering of myelinated axons for all SBEM datasets. Myelinated axons with different thicknesses running along the volumes and organizing bundles with different orientations.

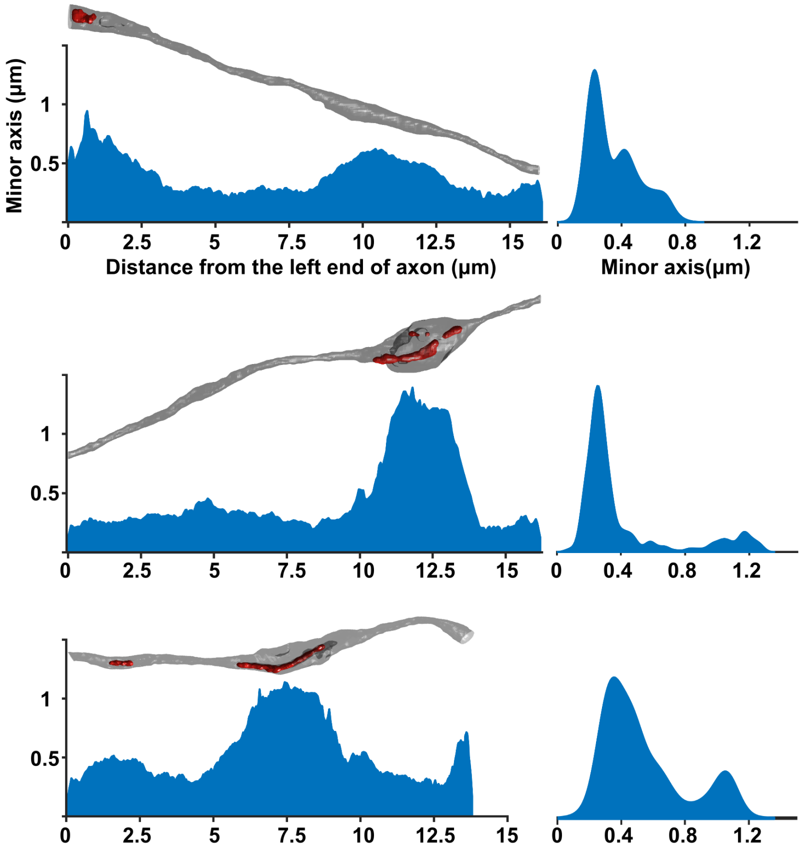

Figure S5. A cross-sectional diameter (minor and major axes or equivalent diameter) is not a constant parameter along a myelinated axon. Accumulation of organelles, such as mitochondria, increases the cross-sectional diameter locally. Therefore, the histogram of cross-sectional diameters for a myelinated axon is more likely to be bimodal.

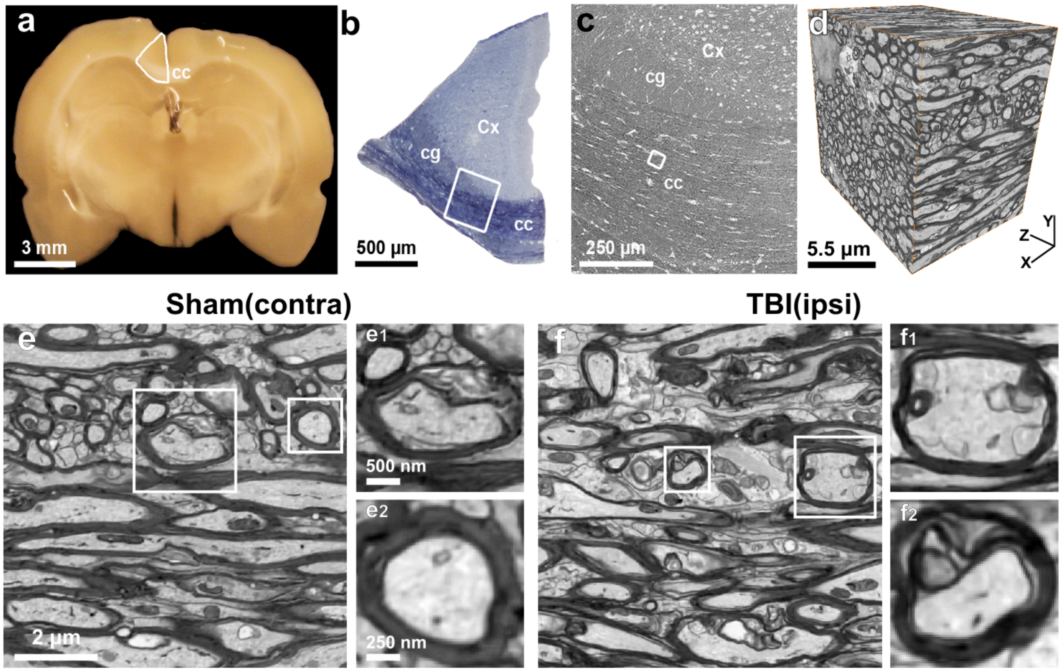

Figure S6. (**a**) A photomicrograph of 1 mm thick coronal section of a sham-operated (Sham-1) rat brain. The white outline shows the selected section for staining, containing part of the corpus callosum, cingulum, and the cerebral cortex. (**b**) A photomicrograph of a semithin section stained with toluidine blue. The white outline shows the block trimmed for SBEM imaging. (**c**) Selection for SBEM imaging (white outline). (**d**) The SBEM volume of the contralateral corpus callosum of Sham-1 dataset. (**e**) A representative 2D image from **d**. (**e1**) and (**e2**) are two representative myelinated axons cropped from **e**. (**e1**) shows myelin delamination. (**f**) A representative 2D image of the ipsilateral corpus callosum of TBI-3 dataset. (**f1**) and (**f2**) are two representative myelinated axons from **f** indicating more frequent myelin delamination as the result of the induced severe injury.

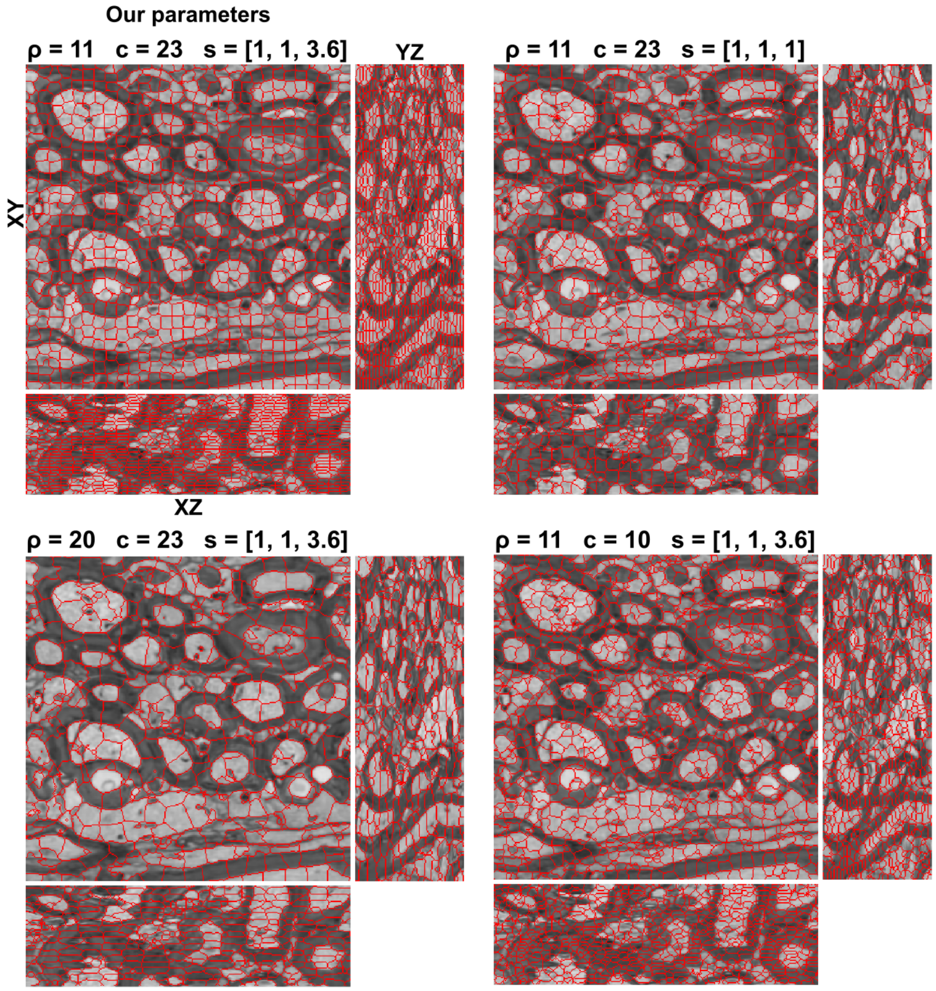

Figure S7. Supervoxel size, compactness and spacing as a function of parameters ρ, c and s for a cropped representative SBEM image. Spacing parameter s allowed us to account for the resolution anisotropy (coarser resolution in z direction) for generating supervoxels. We set spacing parameter in z direction 3.6 times bigger than x and y directions, as the voxel size of Sham-1 dataset was $13.8\,\text{nm} \times 13.8\,\text{nm} \times 50\,\text{nm}$. Increasing ρ enlarges the supervoxels and decreasing c produces more compact and regular supervoxels.

Table S1. Nested ANOVA tables related to Fig. 5 of the equivalent diameter, the minor and major axes and the eccentricity in the contralateral and ipsilateral corpus callosum. Median of cross-sectional measurements was assigned to the lowest level nested ANOVA.

Contralateral

	Source'	'Sum Sq.'	'd.f.'	'Mean Sq.'	'F'	'Prob>F'
	'disease'	0.272317607	1	0.272317607	2.129216996	0.241109601
	'animal(disease)'	0.376745778	3	0.125581926	7.346968256	6.83E-05
EqDiameter	'Error'	28.40861066	1662	0.017093027		
	Total'	29.05767405	1666			
	'disease'	0.174672702	1	0.174672702	1.989201962	0.253765506
	'animal(disease)'	0.258736896	3	0.086245632	6.699700578	0.000170999
MinAxis	'Error'	21.39502184	1662	0.012873058		
	Total'	21.82843143	1666			
	'disease'	0.530654648	1	0.530654648	1.948028395	0.257550582
	'animal(disease)'	0.801977988	3	0.267325996	9.177524644	5.06E-06
MajAxis	'Error'	48.41128985	1662	0.029128333		
	Total'	49.74392248	1666			
	'disease'	0.009072524	1	0.009072524	0.102403085	0.770003651
	'animal(disease)'	0.260552203	3	0.086850734	17.34490004	4.32E-11
Eccentricity	'Error'	8.322095841	1662	0.005007278		
	Total'	8.591720569	1666			

Ipsilateral

	Source'	'Sum Sq.'	'd.f.'	'Mean Sq.'	'F'	'Prob>F'
	'disease'	1.867975755	1	1.867975755	14.39022161	0.029205852
	'animal(disease)'	0.453413312	3	0.151137771	7.085079904	9.93E-05
EqDiameter	'Error'	34.23759862	1605	0.021331837		
	Total'	36.55898769	1609			
	'disease'	0.881901118	1	0.881901118	3.116113668	0.173971226
	'animal(disease)'	1.006357349	3	0.33545245	20.5623783	4.48E-13
MinAxis	'Error'	26.18379907	1605	0.016313893		
	Total'	28.07205753	1609			
	'disease'	4.693643152	1	4.693643152	26.44476127	0.012031724
	'animal(disease)'	0.615855758	3	0.205285253	5.68366584	7.18E-04
MajAxis	'Error'	57.97012697	1605	0.036118459		
	Total'	63.27962588	1609			
	'disease'	0.157228298	1	0.157228298	0.329031221	0.606257915
	'animal(disease)'	1.711910119	3	0.570636706	95.69936956	5.54E-57
Eccentricity	'Error'	9.570302476	1605	0.005962805		
	Total'	11.43944089	1609			

Table S2. Relative volume of the main cellular components to the SBEM volume, expressed as a percentage. Results are dataset-dependent, as the volume fraction that a cell body/process occupies varies among datasets. This affects the volume fraction of the ultrastructure in a dataset, preventing direct cross-analysis between datasets. Therefore, we calculated the aggregate g-ratio and the density of myelinated axons and mitochondria.

	Sham-1		Sham-2		TBI-1		TBI-2		TBI-3	
	Contra	Ipsi	Contra	Ipsi	Contra	Ipsi	Contra	Ipsi	Contra	Ipsi
Myelin (%)	53.5	57.3	39.8	45.3	54.5	44.9	59.5	58	52	51.7
Myelinated axons (%)	26.9	31.4	34.4	25.5	26.9	23.1	23.8	25.4	27	30.6
Number of myelinated axons	367	404	548	234	459	541	650	851	534	418
Mitochondria (%)	3.1	4.2	2.2	1.6	3.7	4.9	3.1	3.5	2.4	2.6
Vacuoles(%)	0.9	1.6	1.4	0.1	0.5	0	1.7	0.3	1.5	1.2
Unmyelinated axons (%)	15.5	9.5	3.7	10.8	9.3	12.8	7.8	13.1	14.5	16.4
Cells (%)	4.1	1.8	22.1	18.4	9.3	19.2	8.9	3.5	6.5	1.3
Aggregate g-ratio	0.58	0.60	0.68	0.60	0.58	0.58	0.53	0.55	0.58	0.61
Density of ultrastructure										
Myelinated axons (μm^{-3})	0.13	0.12	0.14	0.11	0.15	0.13	0.14	0.17	0.14	0.10
Mitochondria (μm^{-3})	0.63	0.58	0.65	1.12	0.22	0.79	0.12	0.16	0.28	0.51

Supplementary video file

The video shows the segmentation of myelin, cell and myelinated axons in the contralateral corpus callosum of sham-1 dataset, respectively. Access/download the supplementary video at: http://www.doi.org/10.5281/zenodo.1459612.
